# Idiopathic Pure Red Cell Aplasia Presenting With Chronic Macrocytosis and Early Relapse: A Case Report and Literature Review

**DOI:** 10.7759/cureus.100985

**Published:** 2026-01-07

**Authors:** Shin Zaw, Diane D Davey, Maria Corrales-Yepez

**Affiliations:** 1 Internal Medicine, College of Medicine, University of Central Florida, Orlando, USA; 2 Pathology, College of Medicine, University of Central Florida, Orlando, USA; 3 Hematology/Oncology, Orlando VA (Veterans Affairs) Medical Center, Orlando, USA

**Keywords:** anemia, bone marrow failure, idiopathic pure red cell aplasia, parvovirus-b19, pure red cell aplasia (prca)

## Abstract

Idiopathic pure red cell aplasia (IPRCA) is a rare immune-mediated bone marrow failure syndrome characterized by isolated anemia and reticulocytopenia. Relapse after remission is incompletely described, and macrocytosis is an uncommon presenting feature that may delay recognition. We report the case of a 69-year-old man with longstanding macrocytic anemia who presented with severe isolated anemia and reticulocytopenia. Extensive evaluation excluded secondary causes, and bone marrow biopsy demonstrated near-complete absence of erythroid precursors, confirming IPRCA. Treatment with concurrent cyclosporine and prednisone resulted in remission within three months. One month after therapy discontinuation, the patient experienced biochemical relapse and achieved re-remission with re-treatment using the same regimen. Immunosuppressive therapy was subsequently discontinued, and the patient remains under active surveillance. To contextualize this case, we performed a Preferred Reporting Items for Systematic Reviews and Meta-Analyses (PRISMA)-guided review of published IPRCA case reports. Cyclosporine-based regimens were associated with the highest remission rates, while relapse and macrocytosis were infrequently reported. This case highlights macrocytosis as a potential atypical presenting feature of IPRCA and underscores the importance of recognizing relapse and ensuring long-term follow-up.

## Introduction

Pure red cell aplasia (PRCA) is a rare bone marrow failure syndrome defined by isolated anemia, severe reticulocytopenia, and near-complete absence of erythroid precursors on bone marrow examination [1]. The estimated annual incidence of acquired PRCA is approximately one per million individuals [1]. Recognized acquired causes include viral infections such as parvovirus B19, autoimmune disorders including systemic lupus erythematosus, thymoma-associated immune dysregulation, and medication-related toxicity, most commonly reported with recombinant erythropoietin and selected anticonvulsants [2]. In a substantial proportion of cases, however, no secondary cause is identified. These idiopathic cases are thought to result from T-cell-mediated immune destruction of erythroid progenitors [2].

Patients with acquired PRCA typically present with symptoms related to severe anemia, including fatigue, dyspnea, and reduced functional capacity, while leukocyte and platelet counts remain preserved [2]. Delayed diagnosis may lead to transfusion dependence, iron overload, and diminished quality of life. Early recognition is therefore essential, as prompt initiation of immunosuppressive therapy can restore erythropoiesis and substantially improve clinical outcomes.

Cyclosporine, with or without corticosteroids, is commonly used as first-line therapy for idiopathic PRCA and yields response rates reported in most series at approximately 70-90%; however, relapse is frequent after tapering or discontinuation, and the optimal duration of therapy and role of maintenance immunosuppression remain uncertain [3].

Although PRCA most often presents with normocytic anemia, macrocytosis is uncommon and may obscure recognition. In addition, relapse following remission, particularly in idiopathic cases, is inconsistently reported in the literature, limiting insight into disease course and long-term management. Here, we describe a patient with IPRCA and longstanding macrocytosis who experienced early relapse after initial remission and achieved a sustained response with re-treatment. We also present a review of published idiopathic PRCA cases to further characterize patterns of presentation, relapse, and outcomes.

## Case presentation

A 69-year-old man with a history of chronic macrocytic anemia (baseline hemoglobin 11.5 g/dL) presented with several days of progressive fatigue, dizziness, and pallor. Colonoscopy performed one year earlier was normal, which excluded occult blood loss as a cause of anemia.

Initial laboratory evaluation demonstrated severe isolated anemia with marked reticulocytopenia and preserved leukocyte and platelet counts (Table 1). Iron studies demonstrated elevated ferritin and transferrin saturation. Evaluation for nutritional deficiencies, endocrine abnormalities, renal dysfunction, autoimmune disease, and hereditary hemochromatosis was otherwise unremarkable.

**Table 1:** Laboratory findings Baseline complete blood count was obtained on admission; additional diagnostic studies were obtained during hospitalization as part of the etiologic evaluation.

Laboratory Parameter	Patient Value	Reference Range
Hemoglobin (g/dL)	6.9	13.5–17.5
Hematocrit (%)	21	41–53
White blood cell count (×10⁹/L)	6.0	4.0–11.0
Platelet count (×10⁹/L)	271	150–400
Absolute reticulocyte count (×10³/µL)	30.3	50–100
Ferritin (ng/mL)	769	30–400
Transferrin saturation (%)	95	20–50
*HFE* mutation testing	Negative	Negative
Folate (ng/mL)	16.4	>4.0
Vitamin B12 (pg/mL)	432	200–900
Mean corpuscular volume	116	80–100
Creatinine	0.8	0.7–1.3
Autoimmune serologies	Negative	Negative

**Figure 1:**
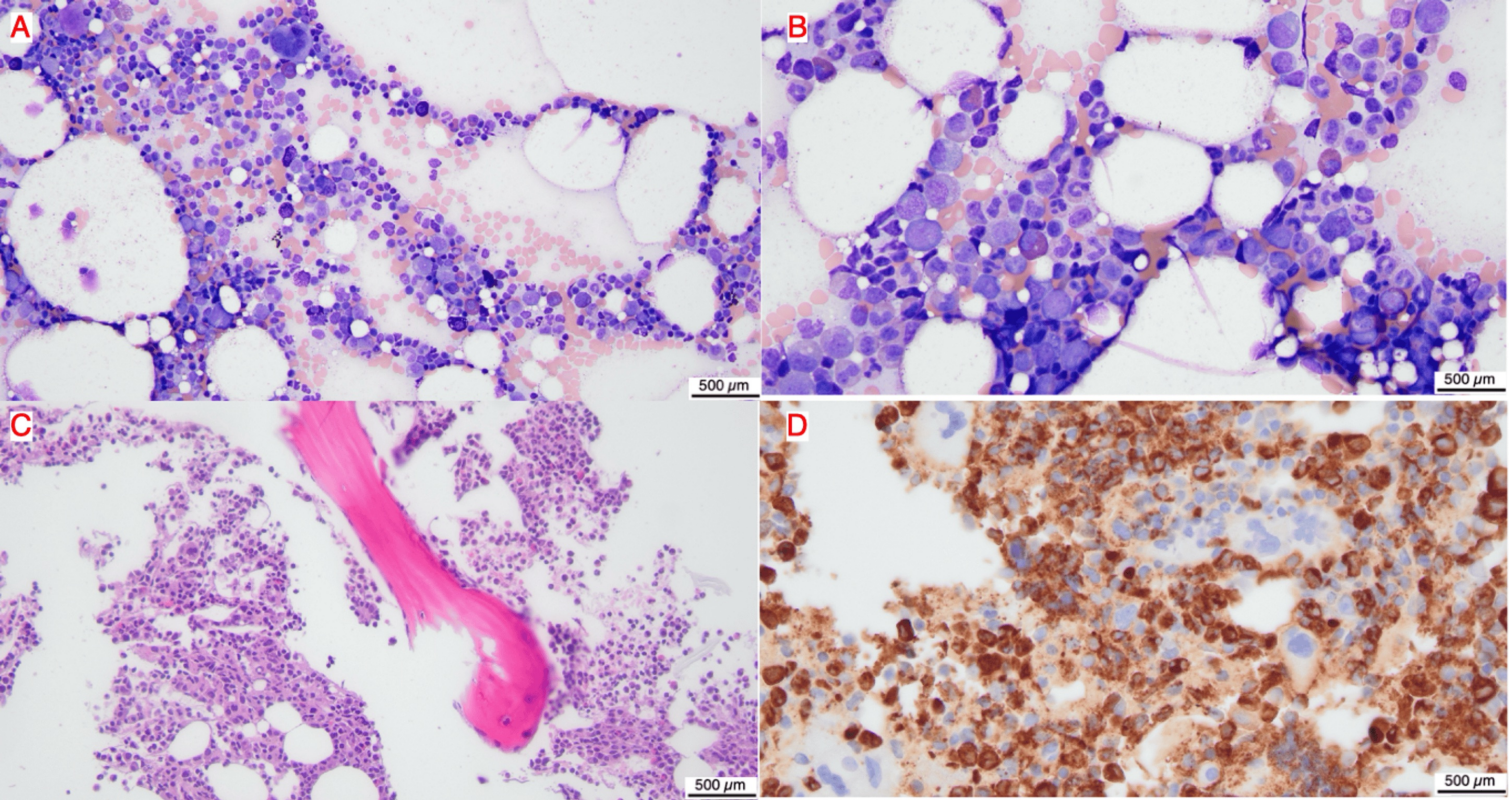
Bone marrow aspirate and core biopsy (A) Wright stain of bone marrow aspirate, 20x magnification, scale bar=500 µm (B) Wright stain of bone marrow aspirate, 20x magnification, scale bar=500 µm (C) Bone marrow core biopsy, hematoxylin and eosin stain, 40x magnification, scale bar=500 µm (D) Bone marrow core biopsy, myeloperoxidase immunohistochemical stain, 40x magnification, scale bar=500 µm

Given the combination of isolated anemia and severe reticulocytopenia, a bone marrow examination was performed. The aspirate and biopsy demonstrated 30-40% cellularity with marked absence of erythroid precursors. Wright-stained aspirate smears (Figures 1A-1B) showed a predominance of granulocytic precursors with scattered lymphocytes, megakaryocytes, and rare mast cells; no erythroid elements were identified. The core biopsy (Figure 1C) confirmed normal cellularity with granulocytic precursors and scattered megakaryocytes, but no erythroid islands. Myeloperoxidase immunohistochemistry (Figure 1D) demonstrated numerous MPO-positive granulocytic precursors, with no clusters of MPO-negative cells identified.

Further workup for secondary causes included a chest CT scan, which showed no thymoma, and viral serologies for Epstein-Barr virus, cytomegalovirus, and hepatitis A, B, and C, all of which were negative. Parvovirus B19 testing was IgG-positive and IgM-negative, indicating prior exposure without acute infection. Serum protein electrophoresis was normal. There was no family history or clinical suspicion for inherited bone marrow failure syndromes; a PRCA/inherited bone marrow failure (iBMF) gene panel was not performed, given the patient’s age and presentation.

Based on the clinical presentation, laboratory findings, bone marrow morphology, and exclusion of secondary causes, a diagnosis of IPRCA was established. The patient received two units of packed red blood cells and was initiated on cyclosporine (300 mg twice daily, target trough 200 ng/mL) and prednisone (20 mg daily). Hemoglobin improved to 12.8 g/dL within three months, and maintenance therapy was continued for an additional three months.

One month after tapering and discontinuation of therapy, the patient experienced biochemical relapse, with hemoglobin declining to 10.6 g/dL despite the absence of symptoms. Re-initiation of the same regimen resulted in a second remission within three months. The patient was subsequently maintained on low-dose immunosuppression with cyclosporine (25 mg twice daily). A repeat bone marrow biopsy performed 20 months later showed no evidence of aplastic anemia. Cyclosporine was discontinued, and the patient remains under active surveillance.

## Discussion

IPRCA is characterized by isolated anemia, severe reticulocytopenia, and age-appropriate marrow cellularity with selective absence of erythroid precursors. The diagnosis is confirmed by bone marrow examination and requires exclusion of secondary causes such as thymoma, autoimmune disease, and viral infections [1,2]. Most patients present with normocytic anemia and respond to immunosuppressive therapy, most commonly cyclosporine with or without corticosteroids, with many achieving remission [3]. Relapse is reported but not consistently documented, and macrocytosis at presentation is uncommon.

In this patient, the diagnosis was established after a bone marrow biopsy confirmed profound erythroid aplasia. Secondary causes were ruled out, including thymoma and active parvovirus B19 infection. Parvovirus B19 selectively infects erythroid progenitor cells, causing lysis and erythroid aplasia; while prior exposure was indicated by IgG positivity, the absence of IgM antibodies and compatible symptoms excluded acute infection. Chronic parvovirus infection is rare and typically occurs in immunocompromised hosts, such as individuals with HIV or those receiving chemotherapy [4]. In acute infection, IgM positivity and high-level viremia on PCR are expected at the hematopoietic nadir [3].

PRCA most often presents with normocytic anemia; however, this patient had longstanding macrocytosis that preceded the development of overt erythroid aplasia [3]. This atypical finding delayed recognition, as macrocytic anemia more commonly prompts evaluation for myelodysplastic syndromes, nutritional deficiencies, liver disease, alcohol use, or endocrine disorders [5]. In this case, these alternative etiologies were carefully excluded, suggesting that the macrocytosis was related to the underlying disease process rather than a secondary cause. The pathophysiology of macrocytosis in idiopathic PRCA is not well defined but may be related to ineffective erythropoiesis from immune-mediated suppression of erythroid progenitors. Ongoing immune injury to early erythroid precursors may disrupt normal red cell maturation, leading to macrocytosis even in the setting of severe reticulocytopenia

Literature synthesis

**Figure 2:**
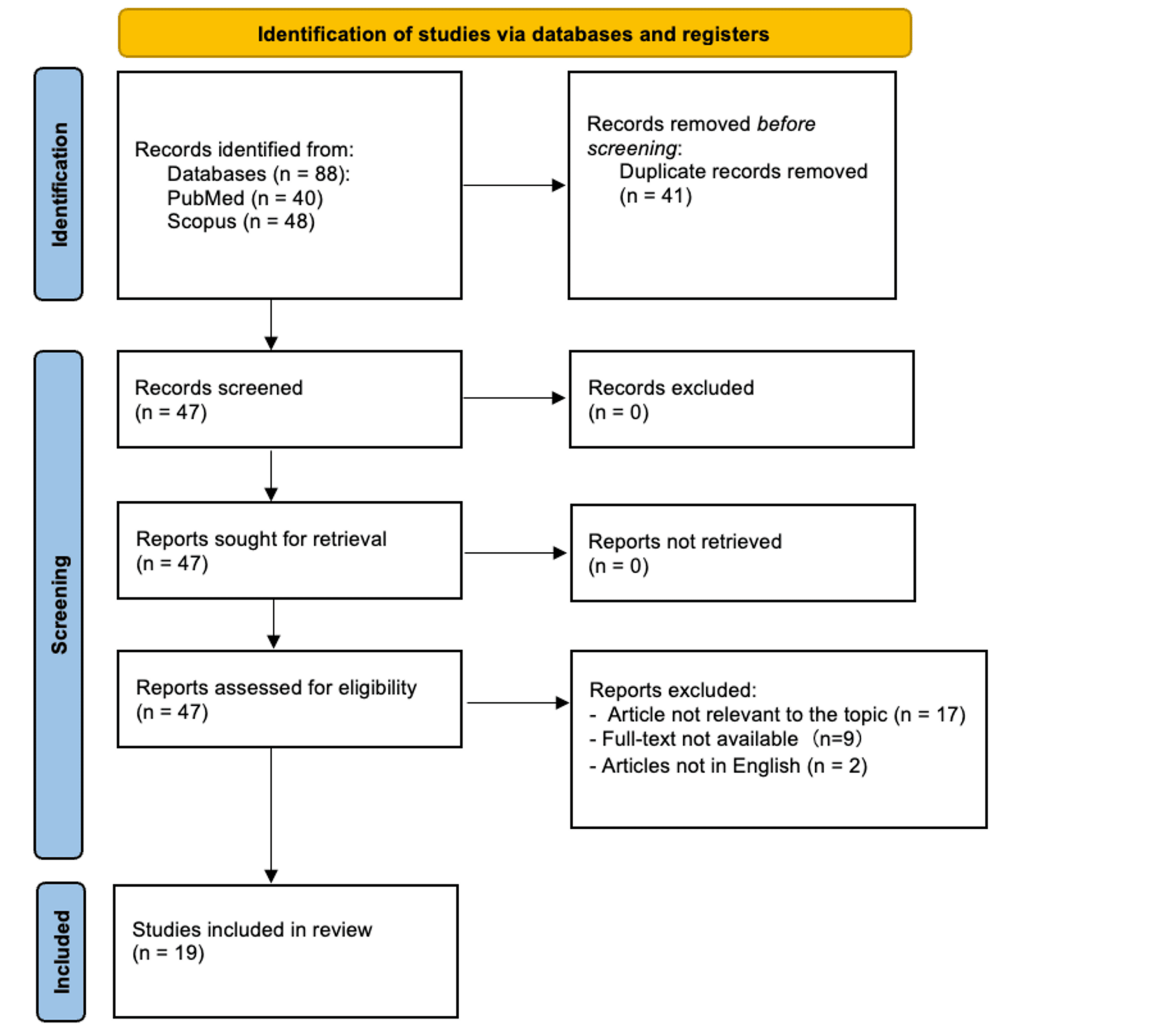
PRISMA flow diagram PRISMA: Preferred Reporting Items for Systematic Reviews and Meta-Analyses

To contextualize this case, we conducted a Preferred Reporting Items for Systematic Reviews and Meta-Analyses (PRISMA)-guided review [6] of published IPRCA case reports in PubMed and Scopus using the search terms “idiopathic” AND “pure red cell aplasia” AND (“case report” OR “case study”), limited to English-language case reports. No restrictions were placed on publication date, as diagnostic criteria for PRCA and first-line immunosuppressive treatment strategies have remained largely consistent over time. Figure 2 depicts the PRISMA flow diagram.

A total of 19 articles met the inclusion criteria, comprising 31 previously reported cases; the present case represents the 32nd. A summary of all 32 cases, including clinical features, treatment approaches, and outcomes, is provided in Table 2.

**Table 2:** Summary of clinical features and responses in case reports of IPRCA (32 patients) CR: complete remission; PR: partial remission; NR: no remission; n/a: not available; M: male; F: female; IPRCA: idiopathic pure red cell aplasia Definitions: Complete remission was defined as normalization of hemoglobin with transfusion independence. Partial remission was defined as a sustained hemoglobin improvement without full normalization and/or reduced transfusion requirements. No remission was defined as lack of meaningful hematologic response or continued transfusion dependence. All therapies were stepwise unless marked with an asterisk (*), which indicates concurrent initiation of therapy

Author(s)	Case Number	Age/Sex	Hemoglobin (g/dl)	White Cell Count (x10^9^/L)	Platelet Count (x10^9^/L)	Associated Disease	Response to therapy (months given)					Outcome
							Prednisone	High-Dose Methylprednisolone	Cyclosporine	Anti-Thymocyte Globulin	Others	
Kwong et al., 1996 [7]	1	76/M	2.7	4.8	227	Nil	NR (1)	-	-	-	-	Transfusion dependent
	2	23/F	3.9	4.4	216	Nil	CR (8)	-	-	-	-	CR
	3	76/F	6.0	5.0	265	Nil	NR (5)	-	-	-	Androgens= NR (5)	Transfusion dependent
	4	56/F	7.3	7.2	519	Nil	CR (3)	-	-	-	-	CR
	5	72/M	3.5	4.0	160	Nil	NR (6)	-	CR (12)	-	-	CR
	6	34/M	6.5	5.2	185	Nil	NR (6)	NR (1)	CR (16)	NR (1)	Androgens= NR (4)	CR
	7	73/F	5.8	4.0	150	Nil	NR (6)	NR (1)	NR (3)	NR (1)	Intravenous gamma globulin= NR (1)	Transfusion dependent
	8	62/F	3.8	4.2	211	Nil	NR (24)	-	CR (9)	-	-	CR
	9	60/M	7.6	4.1	288	Nil	NR (8)	-	PR (3)	-	-	CR
Sivakumaran et al., 1993 [8]	10	58/F	4.3	n/a	n/a	Nil	CR (n/a)	-	-	-	-	CR
	11	68/M	6.0	n/a	n/a	Nil	PR (n/a)	-	-	-	Plasmapheresis = CR (3)	CR
Soda et al., 2001 [9]	12	18/F	3.5	3.5	380	Nil	PR (n/a)	-	PR (n/a)	CR (n/a)	-	CR
Khelif et al., 1985 [10]	13	22/F	3.3	13.3	318	Nil	NR (1)	-	-	-	Plasmapheresis = CR (3)	CR
Jacobs and Wood, 1988 [11]	14	25/F	4.3	2.1	110	Nil	-	-	-	CR (1)	-	CR
Pham et al., 2008 [12]	15	69/M	6.8	5.3	22	Nil	NR (n/a)	-	NR (n/a)	NR (n/a)	Cyclophosphamide = NR (n/a), rituximab = NR (n/a), alemtuzumab = CR (3)	CR
Berlin and Liedén, 1986 [13]	16	57/M	6	Normal	Normal	Nil	NR (1)	-	-	-	Leucapheresis, plasmapheresis, and cyclophosphamide = CR (1)	CR
Au et al., 2005 [14]	17	35/M	n/a	n/a	n/a	Nil	-	-	PR (1)	PR (1)	Monoclonal antibody = CR (1)	CR
Ramadan et al., 2005 [15]	18	43/M	4.2	5.1	497	Nil	CR (n/a)	-	-	-	-	CR
Ahn et al., 2001 [16]	19	55/M	6.7	2.5	187	Nil	PR (32)	NR (n/a)	NR (n/a)	PR (4)	Fludrabine = CR (1)	CR
Gangat et al., 2022 [17]	20	74/F	5	n/a	n/a	Nil	PR (n/a)	-	PR (n/a)	PR (n/a)	Rituximab, alemtuzumab, cyclophosphamide, bortezomib, eltrombopag, danazol = PR (n/a)	Transfusion dependent
Vo et al., 2020 [18]	21	77/M	6	6.8	445	Nil	CR (21)	-	-	-	-	CR
Al-Issa et al., 2015 [19]	22	63/M	5.6	5	300	Nil	NR (n/a)	-	CR (10)	-	Rituximab = NR (n/a)	CR
Yildirim et al., 2013 [20]	23	20/F	4.6	7.5	429	Nil	-	-	CR (3)	-	Mycophenolate mofetil, Tacrolimus = NR (2)	CR
Okada et al., 1994 [21]	24	43/M	6.8	8.8	530	Nil	-	-	-	-	High dose erythropoietin = NR (6)	NR
Mant, 1994 [22]	25	21/F	6.7	7.2	332	Nil	-	-	-	-	Intravenous gamma globulin = CR (n/a)	CR
Chang et al., 1978 [23]	26	53/M	7.8	8.4	210	Nil	NR (n/a)	-	-	-	Androgens = NR (n/a), Oxymetholone = CR (n/a)	CR
Yamada, 1999 [24]	27	43/M	11.1	5.6	425	Nil	-	NR (3)	CR (1)	-	Androgens = NR (12)	CR
	28	40/F	3.5	5.6	508	Nil	-	-	CR (9)	-	-	CR
Kawano et al., 2013 [25]	29	1/M	5.3	n/a	n/a	Nil	CR (2)	-	-	-	-	CR
	30	91/F	8.2	n/a	n/a	Nil	-	-	CR (2)	-	-	CR
	31	32/F	4.8	n/a	n/a	Nil	CR (2)	-	-	-	-	Patient had relapsed and was treated with combination of cyclosporine and prednisone and is in re-remission.
Current case	32	69/M	6.9	6.0	271	Nil	CR (3)*	-	CR (3)*	-	-	Patient was re-treated with cyclosporine and prednisone, resulting in re-remission. Immunosuppressive therapy was later discontinued, and he is currently undergoing active surveillance.

Across these cases in Table 2, the median age was 55 years, with 17 of the 32 patients (53.16%) being male. Cyclosporine-based regimens were associated with the highest rates of complete remission, observed in 10 (31.25%) patients. Prior to inclusion of the present case, relapse was explicitly reported in only one IPRCA case (case 31), suggesting that recurrence may be uncommon or, more likely, underreported in the literature [25]. Among the cases summarized in Table 2, treatment was initiated in a stepwise manner, typically beginning with corticosteroids and escalating to cyclosporine or other immunosuppressive agents in the setting of inadequate response or relapse. In contrast, our patient was treated with concurrent cyclosporine and corticosteroids at diagnosis, resulting in rapid hematologic improvement. The same regimen was effective when reinitiated after early relapse, mirroring the treatment strategy and outcome reported in case 31 [25].

## Conclusions

IPRCA should be considered in patients with isolated anemia and reticulocytopenia, even when macrocytosis is present. This case shows that relapse can occur soon after remission, as our patient relapsed within one month of stopping therapy, highlighting the need for close follow-up during this period. Re-treatment with cyclosporine and corticosteroids was effective. Our review of the literature suggests that relapse is infrequently reported, likely due to variable follow-up, and underscores the importance of ongoing surveillance in patients with IPRCA. More consistent reporting of relapse timing, treatment duration, and response to re-treatment may improve understanding of disease course and help inform follow-up and management strategies.
